# Prediction of dementia following traumatic injury with risk score (the DEMTIS): a multivariable prediction model development study based on Hong Kong electronic health records

**DOI:** 10.1192/j.eurpsy.2025.483

**Published:** 2025-08-26

**Authors:** C. S. Fung, H. F. Chung, S. H. Cheung, N. Dong, I. T. K. Hui, C. S. M. Wong, W. C. Chang, P. W. Cheng

**Affiliations:** 1 Department of Psychiatry; 2 Department of Psychology; 3 School of Public Health, The University of Hong Kong, Hong Kong, Hong Kong

## Abstract

**Introduction:**

Patients following traumatic injury (TI) are at increased risk of developing dementias, yet an efficient, validated screening instrument is lacking.

**Objectives:**

In the current study, we developed the Dementias following Traumatic Injury Screening (DEMTIS) score, a brief post-TI dementias screening tool.

**Methods:**

We identified 270,843 electronic health records from Hong Kong patients admitted for TI between 2001 and 2021. The records were randomly split into training (80%; n=258,739) and testing cohort (20%; n=50,883). The DEMTIS was developed based on a backward stepwise multivariate Cox proportional hazard model predicting first-ever dementia diagnosis. Competing risk survival analyses were used to predict the risk of Alzheimer’s disease (AD) and vascular dementia (VD), while taking the risk of other dementias into account. Model discrimination of the three scores was evaluated using concordance statistics (c-statistic) calculated as the area under the receiver operating characteristic curve. Statistical significance was set at p<.01.

**Results:**

The 5-year, 10-year, and 20-year risk of all-cause dementias following TI was 2.1% (95% CI 0.020-0.021), 3.8% (95% CI 0.037-0.039), and 6.5% (95% CI 0.063-0.066), respectively. The final model included sex, TI characteristics, physical covariates, history of mood and anxiety disorders, and cerebral degenerative disease (See Table 1). The population mean of DEMTIS was 59.45 (SD=21.29). The optimal threshold of DEMTIS predicting dementia was determined at 75 using the closest top left rule. Individuals at high risk (DEMTIS≥75) were associated with a 6.0% (95% CI 0.059-0.061) risk of dementia in 5 years, whereas those at low risk were associated with a 0.5% (95%CI 0.004-0.005) risk (see Figure 1; Figure 2). The model predicting 5-year dementia has an overall c-statistic of 0.835 (95% CI 0.832-0.839) in the testing data (see Figure 3). We further developed risk scores for 5-year AD and VD based on the findings from competing risk models; the c-statistics of model for AD and VD are 0.857 (95% CI 0.844-0.871) and 0.837 (95%CI 0.821-0.853) respectively.

**Table 1:** Calculation of the DEMTIS

Variables	DEMTIS	DEMTIS-AD	DEMTIS-VD
Sex			
Male	0	0	0
Female	2	1	1
Age of TI	1	1	1
Position of TI			
Head injury	1	-1	2
Torso	1	2	1
Upper Limb	2	2	2
Lower Limb	0	0	0
Fracture types		NA	
Opened	-1		-1
Closed	0		0
Unspecified	-1		-1
Hypertension			
Yes	2	-1	3
No	0	0	0
Hyperlipidemia			
Yes	2	-1	2
No	0	0	0
CCI	-1	-1	2
Cerebral degenerative disease			
Yes	3	3	3
No	0	0	0
Mood and anxiety disorder			NA
Yes	2	2	
No	0	0

**Image 1:**

**Figure:**
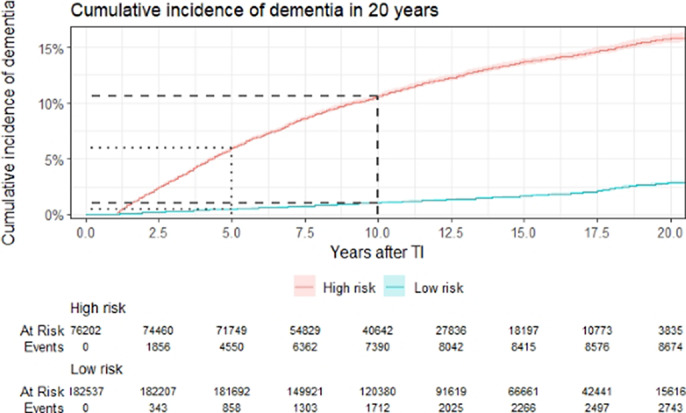

**Image 2:**

**Figure:**
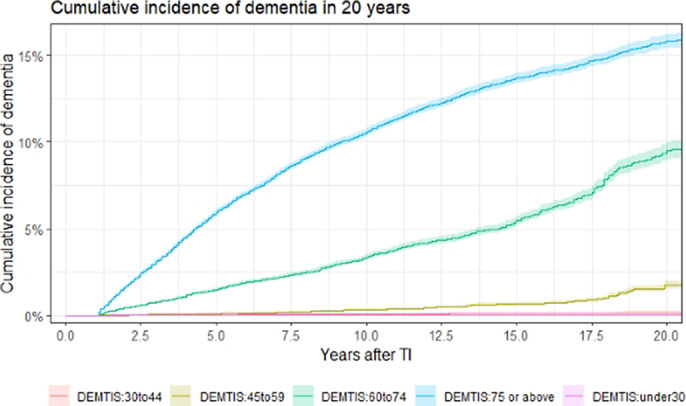

**Image 3:**

**Figure:**
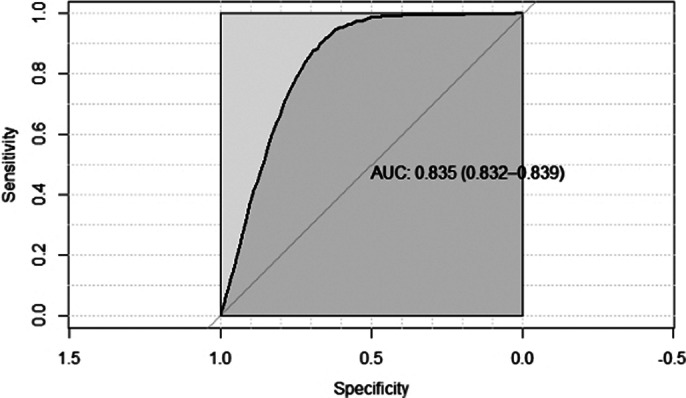

**Conclusions:**

As a novel, easily accessible screening instrument, DEMTIS can identify patients at elevated risk of dementia following TI. It assists clinicians in evaluating patients’ risk of dementia and providing personalized care.

**Disclosure of Interest:**

None Declared

